# Activated Membrane Patches Guide Chemotactic Cell Motility

**DOI:** 10.1371/journal.pcbi.1002044

**Published:** 2011-06-30

**Authors:** Inbal Hecht, Monica L. Skoge, Pascale G. Charest, Eshel Ben-Jacob, Richard A. Firtel, William F. Loomis, Herbert Levine, Wouter-Jan Rappel

**Affiliations:** 1 Center for Theoretical Biological Physics, University of California San Diego, La Jolla, California, United States of America; 2 School of Physics and Astronomy, Raymond and Beverly Sackler Faculty of Exact Sciences, Tel-Aviv University, Tel Aviv, Israel; 3 Cell and Developmental Biology, Division of Biological Sciences, University of California San Diego, La Jolla, California, United States of America; 4 Department of Physics, University of California San Diego, La Jolla, California, United States of America; University of California San Diego, United States of America

## Abstract

Many eukaryotic cells are able to crawl on surfaces and guide their motility based on environmental cues. These cues are interpreted by signaling systems which couple to cell mechanics; indeed membrane protrusions in crawling cells are often accompanied by activated membrane patches, which are localized areas of increased concentration of one or more signaling components. To determine how these patches are related to cell motion, we examine the spatial localization of RasGTP in chemotaxing *Dictyostelium discoideum* cells under conditions where the vertical extent of the cell was restricted. Quantitative analyses of the data reveal a high degree of spatial correlation between patches of activated Ras and membrane protrusions. Based on these findings, we formulate a model for amoeboid cell motion that consists of two coupled modules. The first module utilizes a recently developed two-component reaction diffusion model that generates transient and localized areas of elevated concentration of one of the components along the membrane. The activated patches determine the location of membrane protrusions (and overall cell motion) that are computed in the second module, which also takes into account the cortical tension and the availability of protrusion resources. We show that our model is able to produce realistic amoeboid-like motion and that our numerical results are consistent with experimentally observed pseudopod dynamics. Specifically, we show that the commonly observed splitting of pseudopods can result directly from the dynamics of the signaling patches.

## Author Summary

Different types of cells are able to directionally migrate, responding to spatially-varying environmental cues. To do so, the cell needs to sense its environment, decide on the correct direction, and finally implement the needed mechanical changes in order to actually move. In this work we study the relation between the sensing-signaling system and the mechanical motion. We first show that membrane protrusions which drive the overall translocation occur exactly at the same locations at which membrane-bound signal-transduction effectors accumulate. These high concentration areas, also termed “patches”, exhibit interesting dynamics of disappearing and reappearing. Based on these findings, we develop a mathematical-computational model, in which membrane protrusions are driven by these membrane “patches”. These protrusions are then coupled to other cellular forces and the overall model predicts motion and its relationship to shape changes. Using our approach, we show that several observed features of cellular motility, for example the splitting of the cell tip, can be explained by the upstream signaling dynamics.

## Introduction

Directional cellular migration is a widely observed phenomenon, ranging from mammalian cells to unicellular eukaryotes to bacteria. During development, as well as in mature organisms, cells respond to environmental cues and migrate to distant sites to perform different tasks, such as wound healing or immune response [1]. In other cases, cells respond to a nutrient gradient and migrate towards a food source [2], [3], or aggregate to form a multi-cellular slug [4], [5]. Directional motion according to external cues, known as chemotaxis, is typically controlled by signaling processes in the cell. Through signal transduction pathways, the external stimulation leads to internal symmetry breaking and to the formation of a distinct front and back. This sensing step is then coupled to cell mechanics, which is also governed by signaling processes which are highly conserved between different organisms [6].

In the last decade, many studies have been devoted to the characterization of different signaling components and systems in different organisms (see e.g. [7]–[9]). Other studies, both theoretical and experimental, have dealt with the biophysics of cellular motion including such aspects as actin polymerization, adhesion and myosin-based contraction [10]–[14]. However, an understanding of the coupling between the two systems – directional sensing and motility mechanics –is still incomplete, both from the experimental and the theoretical points of view. A modeling study of this coupling was undertaken in ref. [13], but from a perspective that does not build on observed correlations between these two parts of the overall chemotactic response. Yang *et al.* [15] used the level set method to link cell deformations with signaling events including PIP3 localization to calculate the pressure profile in a cell. However, their model was unable to predict experimentally observed cell shapes, probably because it did not take into account the complex signaling dynamics. In this paper we study how the signaling pattern and dynamics influence macroscopic features of cellular shape and motion, by using both experimental data and computational modeling. Specifically, we show that several experimental observations of cell motion can be explained by a better understanding of the spatio-temporal aspects of the aforementioned coupling.

In the social amoeba *Dictyostelium discoideum*, a large number of the signaling components have been identified through extensive genetic and biochemical investigations, along with their spatial intra-cellular distribution relative to an external gradient [7], [16], [17]. This distribution is usually non-uniform with several components located at the front while others are concentrated at the back of the cell [18], [19]. The cell motion is then accomplished by membrane protrusions at the front of the cell, along with retraction at the back. These protrusions are generated through the polymerization of actin filaments while the retraction is associated with cortical tension generated by actin-myosin interactions [20], [21]. For amoeboid cells, the protrusions take the form of pseudopods with finite life-times, leading to repeated cycles of extension and retraction.

One of the earliest measurable signaling events is the appearance of activated Ras, RasGTP, to the front of the cell [22]. This is then followed by the recruitment of other signaling molecules with a number of feedback loops [23]. Such experiments are typically carried out by exposing cells to a steep gradient originating from a pipette and do not address the subsequent motion of the cell. When exposed to a uniform stimulus, cells display a number of membrane “patches” in which the concentration of a signaling molecule is greatly increased. These patches have been implicated in the formation of pseudopods [24], [25] and RasGTP has been shown to co-localize with the site of F-actin polymerization in both chemotaxing cells and in cells undergoing random motility [22], [26]. Furthermore, it has been reported that RasGTP can drive localized actin polymerization via PI3K [27]. Recently, a number of features of chemotactic cell motion, including the rate of pseudopod formation, the distribution of *de novo* pseudopods and their persistence have been studied quantitatively [28]–[30]. This analysis revealed that the rate of formation of pseudopods is roughly independent of orientation of the cell with respect to the shallow gradient. Furthermore, it was argued that new pseudopods are not always located in the direction of the highest receptor occupancy, inconsistent with a deterministic “chemical compass” model [31], [32]. In fact, these experiments have been taken to imply the existence of a specialized tip splitting mechanism in which the location of a new pseudopod is highly correlated with the location of the current pseudopod from which it splits off.

Because the coupling of the directional sensing pathways to the motility machinery is currently not well understood, it has been difficult to develop detailed mathematical models that can simulate realistic cell motion. Most models to date have addressed distinct parts of motility, including retraction and protrusion [33], but are unable to describe the entire motility process; other models use ad-hoc rules to describe the motion [11], [12]. What has been lacking is a model that couples elements of the sensing machinery to cell motility.

Part of the challenge of developing models has been the lack of experimental data that reliably identify membrane protrusions mechanisms. Many experiments have been performed in assays where a thin horizontal subsection of the cell was visualized by confocal microscopy. Chemotaxing cells, however, can extend into the vertical direction. This vertical extent makes it difficult to quantify the correlation between the localization of signaling components and membrane extensions. In this paper, we examine the localization of RasGTP by GFP-tagged Ras binding domain (RBD). RBD-GFP intensity was measured along the membrane of *Dictyostelium* cells moving in a chemoattractant gradient and correlated with pseudopodal protrusions. This was done with cells restricted in the vertical direction such that fluorescent patches at the membrane could be visualized in a single confocal section and membrane protrusions could be quantitatively measured. In the first set of experiments, we used the well-established under-agar assay in which cells must lift a thin layer of agar as they move [34], while in the second set of experiments, we employed a microfluidic device in which the cells are constrained by the height of the chamber [35]. We used the results from these experiments to perform a quantitative analysis of the spatial correlation between signaling components and pseudopod extensions and found a strong spatial correlation between patches of RasGTP and membrane protrusions.

On the basis of these new experimental data, we develop a mathematical model for cell motion in which cell protrusions are driven by patches of an activator, qualitatively similar to the observed RasGTP patches at the front of chemotaxing cells. Our model incorporates a set of mechanisms that allow for the simulation of cell motion under a variety of experimental conditions and is studied here for the specific case of patch-driven chemotactic response in a static gradient. We show that our model produces realistic amoeboid-like motion and can demonstrate the effects of gradient steepness, cortical tension, and polarity on the cell shape and motion. Our model shows that the patch dynamics of membrane bound activators result in an apparent tip splitting behavior and that therefore an explicit splitting mechanism is not needed. Specifically, a patch in our model is stable and will not bifurcate into two or more spatially distinct patches before disappearance, as is the case in several physical systems [36]. Furthermore, we show that the apparent process of the cell “choosing” the better-oriented pseudopod [37] is simply an outcome of the disappearing-reappearing dynamics of the activator patches. Finally, we show that the results of automated pseudopod detection algorithms need to be carefully interpreted.

## Results

### Experimental Results

**Figure 1:**
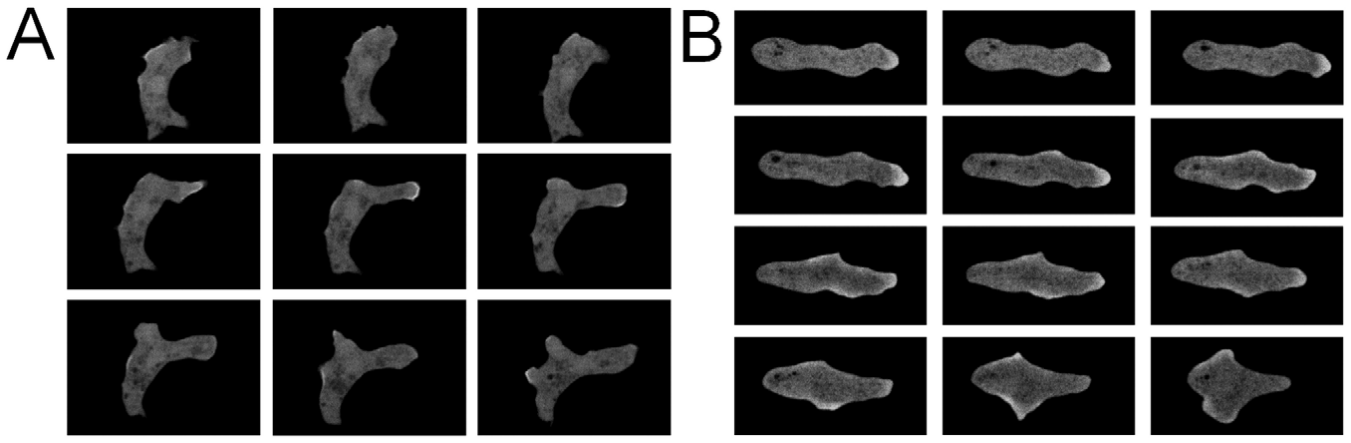
*Dictyostelium* cells with RBD-GFP. A series of snapshots from the under-agar experiment (a) and from the microfluidic experiment (b). Time between frames is 10 seconds.

The Ras binding domain from human Raf1 binds strongly to RasG in the GTP bound form [22], [26], [27], [38]. We used RBD-GFP to track the localization of RasGTP at the cell membrane in chemotaxing *Dictyostelium* cells at 2 second intervals. Figure 1 shows several snapshots of these cells in both the under-agar assay (a) and the microfluidic assay (b). In both experimental setups, the vertical dimension of the cells was restricted (for more details see Methods), which ensured that most of the cell body remained in the focal plane of the microscope during its motion. In the microfluidic device, cells entered cross chambers only 2 µm high that connected parallel channels carrying buffer on one side of the cross chambers and 100 nM cAMP on the other. The length of the cross chambers varied from 650 µm to 100 µm thereby generating gradients of different steepness.

The cell speed in the under-agar assay, as well as the microfluidic devices, was found to be 8–10 µm/min. The chemotactic index (CI), defined as the ratio of the distance traveled in the direction of the gradient and the total distance, was 0.71 to 0.94 for cells in the microfluidic devices. It was not possible to compute a CI for the under-agar experiments since the precise direction of the gradients is not known.

We quantitatively compared the location of RBD-GFP patches and the location of membrane protrusions. Patches were detected using a global threshold for filtering background intensity and protrusions were detected by comparing the membrane location in successive frames (see Methods). The location of a patch in each frame can be defined by an angle *θ* between an arbitrary axis and the line connecting the center of the patch and the center of the cell. A similar angle *ϕ* can also be defined for the location of the pseudopod. An example of this analysis is presented in Figure S1 in the Supporting Information section.

To test the spatial correlation between the locations of patches and protrusions, we define a correlation function between a patch and a protrusion at frame *i* as
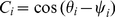
(1)This correlation function takes on values between −1, corresponding to anti-correlated patch and pseudopod locations, and 1, corresponding to a patch location that coincides exactly with the pseudopod location. If patches and protrusions are completely uncorrelated this correlation function should average to zero (data not shown).

**Figure 2:**
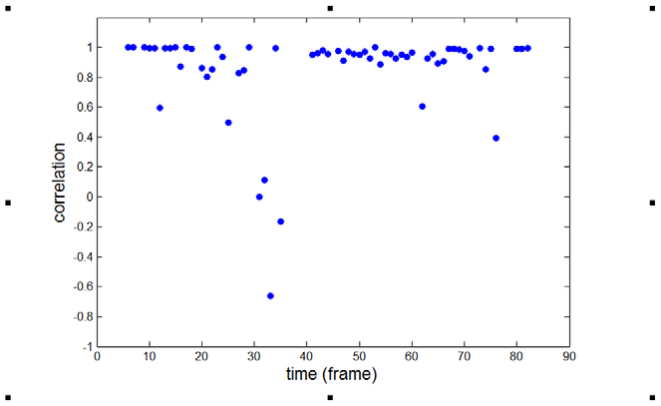
Spatial cross correlation between patches and protrusion (**Eq. 1**) for a single cell in the microfluidic device as a function of the frame number.

The correlation function of Eq. (1), for a particular cell in the microfluidic device, is shown in Figure 2 and remains close to the maximal value of 1 for most frames. We have analyzed three cells in the under agar experiment (305 frames, 297 of which showed both a patch and a pseudopod). We found an average correlation function of 0.83, 0.87 and 0.89 for co-localization of RasGTP patches and membrane protrusions. In the microfluidic device we analyzed eight cells, totaling 3421 frames of which 2289 showed both a patch and a pseudopod. Taking the data from all frames in both experiments that contain both a patch and a protrusion we found an average correlation function of 0.90 (±0.04). This correlation analysis implies a close relationship between protrusions and RasGTP patches: a new protrusion is accompanied by membrane-localized RasGTP accumulation in the same place in space. Furthermore, the fact that the correlation function for both assays is similar suggests that this relationship is independent of the experimental details. We have also tested five cells under uniform stimulation of cAMP (100 nM) and found an average correlation of 0.9 (±0.05). This indicates that the RasGTP-protrusion correlation is not specific to a gradient sensing process.

The measured strong correlation is consistent with a causal relationship in which a RasGTP patch almost always leads to a membrane protrusion. Previous experiments have demonstrated that Ras activation mediates leading edge formation, through activation of basal PI3K and other Ras effectors required for chemotaxis [22]. It was also shown that mutants with defective RasG exhibited a loss of directionality and severe loss of movement [22]. In this work, we focus on the spatial correlation, demonstrating that activated Ras localization and pseudopod formation occur at the very same location in the cell.

### Computational Motility Model

Based on the abovementioned results, we developed a computational motility model in which protrusions are generated by membrane patches of a putative chemical activator. The goal of the model is to allow for the study of the effects on cellular morphology of localized, transient protrusion forces, assumed to originate from the signaling system downstream of the patch dynamics. To do this requires embedding a patch generation mechanism into a full cellular mechanics simulation. In the absence of a complete understanding of all the relevant biophysical effects at the whole cell level, we opted for creating a relatively simple simulator, taking many experimental movies both from our own lab and from other groups (see [30], e.g.) as guidance. Later, we will discuss in detail which aspects of our results should be insensitive to some of the details of the mechanical model.

Following the aforementioned strategy, our motility model consists of two coupled modules: the first module contains a mechanism that creates transient localized patches while the second module describes the actual motion of the cell. We will consider a two-dimensional cell and will represent its membrane by a set of nodes. For the first module, we choose our recently developed excitable reaction-diffusion model which contains an activator field *a* and an inhibitor *b* [39]. Even though our model is not formulated at the level of specific biochemical components we can nonetheless use the activator *a* to mimic the observed behavior of activated Ras patches, so that their influence on downstream motility can be tested. The equations governing these fields can be written as
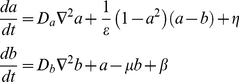
(2)where *D_a_* and *D_b_* are the diffusivities of *a* and *b*, respectively, *ε*, *β*, and *μ* are constants and *η* is a noise term. This term is taken from a uniform distribution in the range [−1,1], but, to avoid overwhelming the system by simultaneous excitation of many coupled points, only a small fraction of the points (0.001–0.01%) are randomly given a non-zero noise term [39]. Such a noise pattern can be generated by feeding Gaussian white noise into a nonlinear excitable process (data not shown). Such processes have been directly demonstrated in genetic networks [40], [41]. In our previous work, we have shown that this model is excitable for a certain range of parameter values and that the inclusion of the noise term leads to the spontaneous formation of domains of high *a*. Due to the excitable nature of the model, these *a*-patches spontaneously disappear, followed by the appearance of new patches, similar to the observed RasGTP dynamics in our experiments and to the dynamics of PIP3 patches [24]; this has been discussed in detail elsewhere [39].

It should be noted that detailed and precise modeling of Ras dynamics is beyond the scope and purpose of this work. Once again, our goal is to test how the patch dynamics, and specifically its come-and-go nature, influence the macroscopic cell shape and motility. For this purpose, we only need a system that creates patches of one of the species, which can then be used as an activator for downstream processes. In fact, one can completely replace the patch dynamics by an artificial process which puts patches in by hand with the measured distributions, and recover all of our results (data not shown).

**Figure 3:**
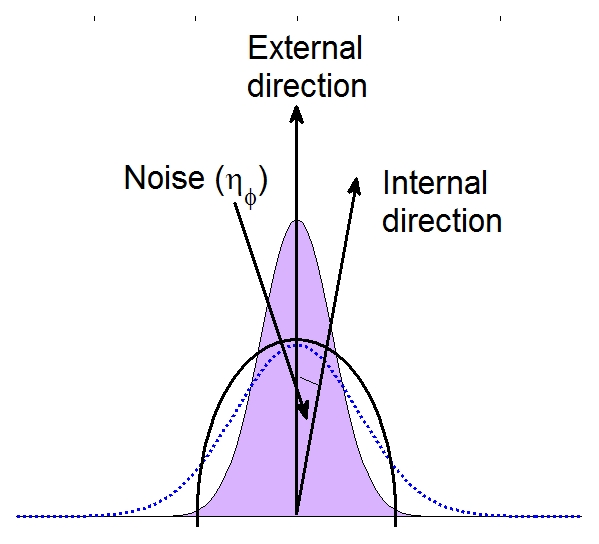
Directionality mechanism in the computation model. The cell membrane is shown (circular line) together with the external direction, determined by the external gradient. The internal cellular direction, the internal compass, is chosen from a Gaussian distribution with its peak in the direction of the gradient and with a width that depends on the gradient steepness: steep gradients results in a narrow distribution (shaded area) while shallow gradients give rise to a wider distribution (dotted line).

The excitability of the system is controlled by the parameter *β* : below *β*<0.6 the system is highly excitable while above this value the excitability is significantly reduced. Thus, varying this parameter along the cell boundary determines the rate of patch formation and choosing the front of the cell to be excitable while the back of the cell is unexcitable will lead to patch formation concentrated at the cell's front. Here, we do not explicitly concern ourselves with modeling the gradient sensing mechanism that detects the external chemical concentration field and determines *β*. Indeed, how a cell determines its front has been the subject of many theoretical studies [42], [43]. Here, we directly assume that front determination is accomplished through the formation of an internal compass. The direction of this compass is determined by the receptor occupancy and is therefore dependent on the external gradient direction. Specifically, we choose the internal compass direction, *ϕ_int_*, to be the external direction *ϕ_ext_* plus some added noise:
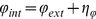
(3)The term *η_ϕ_* represents all the possible fluctuations in the directional sensing process and is drawn from a Gaussian distribution with zero mean and width *σ* (see Figure 3). We assume that the width of the noise distribution is inversely proportional to the steepness of the gradient such that the directional sensing process is more accurate for steeper gradients (Figure 3) as is reflected in the increased chemotactic indices of cells responding to steeper gradients [44]. The front of the cell is then chosen to be the point on the membrane that is closest to the direction of the internal gradient *ϕ_int_*. Once the angle is determined, *β* is chosen to be peaked around the front with a width that inversely depends on a (dimensionless) polarizability parameter *p*. This parameter *p* determines how abruptly *β* changes with the distance from the cell front and, hence, has an impact on the width of the excitable zone on the membrane. A high value of *p* corresponds to cells with a smaller width of the excitable zone and, thus, to more polarized cells, while a low value of *p* represents a larger excitable membrane zone and less polarized cells. The precise form of the excitability along the membrane is given in the Supporting Text S1.

The noise distribution width *σ*, the polarizability parameter *p* and the excitability *β* represent different aspects of the cellular response. The response of the cell depends on its polarization level: in relatively symmetric cells characteristic of early developmental stages, projections extend all along the cell's periphery, while in polarized (elongated) cells projections only extend at the cell's front [45]. This change is represented by *p*, which is an internal property of the cell and is hence independent of the external conditions. The polarization and gradient are connected to the patch formation mechanism through the parameter *β* (see also [39] and the Supporting Text S1 and Figure S2). As mentioned above, this parameterization is aimed at realistically describing the signaling system, so that the influence of different signaling behaviors on the cell motility can be tested.

The second module is responsible for cell motion through the definition of a force on each node, taken to be normal to the cell membrane:
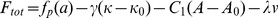
(4)In this equation, the first term results from the localization of *a* and couples the signaling module to the motility module. Specifically, motivated by our experimental results, this protrusion force is assumed to depend on the concentration of the activator *a* (describing RasGTP) in the first module. For simplicity, we have chosen a simple linear dependence as detailed in the Supporting Text S1. Using other forms, including those with a non-linear dependence, yielded essentially similar results.

In the actual cell, the relationship between the chemical driving and eventual actin polymerization leading to protrusion forces is rather complex. Under most conditions, our cell simulator is able to ignore all of these complications and get by with the simplest possible linear relationship. However, we show in Video S1 and in Figure S3a that for the case of driving the cell with *two* strong sources on opposite sides of the cell (see for example [30]) that this model is unable to capture the fact that pseudopods must eventually compete with each other and only one can win in the long run. We have therefore added one extra part to this patch chemical – protrusion force relationship, making it depend on a global resource *G(t)* which is consumed by the pseudopod construction process (see for example [45], where the authors state that cytoskeletal or membrane components are probably limited, causing the cell to occasionally “freeze”). Video S2 and Figure S3b show that, indeed, adding this effect yields the observed cell behavior. The details of how *G* is dynamically determined are discussed in the Supporting Text S1. For the case of chemotactic motion to a simple gradient, the case of primary interest here, this feature is relatively unimportant (see later).

The second term in the right side of Eq. (4) describes the cortical tension, which depends on the local curvature *κ*. *γ* represents the membrane rigidity, with higher values of *γ* corresponding to more rigid membranes. *κ_0_* is the spontaneous curvature of the cell, which is the equilibrium curvature when the total force is zero, namely 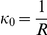 for a circular cell of radius *R*. Due to the differences in the acto-myosin cortex structure around the cell versus the protrusion area, we take *γ* to depend on position along the cell membrane. Recent experiments [46]–[48] have revealed that the tension is higher at the back, where presumably myosin bundles and crosslinks the cortical actin layer, as opposed to the front of the cell; thus we choose the back part of the cell, defined as the portions of the membrane for which *a<0*, to have a cortical tension (*γ_1_*) that is about twice as high as the cortical tension (*γ_2_*) in the front part of the cell where *a>0*. In addition, we have empirically discovered that in order to produce pseudopods with large aspect ratio, i.e. long and narrow, and to get the “valleys” between pseudopods to have a reasonable shape, we need to allow regions of the membrane with negative curvature to have a value *γ_3_* that is smaller yet. A possible origin of this effect lies in that we are using a two-dimensional model to describe a three-dimensional cell (albeit moving within a limited three dimensional space). The tension force in 3D should of course be proportional to the total curvature and it might be the case that negative in-plane curvature tends to cancel the positive out-of-plane curvature, resulting in small net effect. In the Supporting Information (Text S1 and Figure S4) we show how this effect modifies cell shape dynamics and makes them more “biological”. It is important to note though that this additional assumption is *not* necessary in order to obtain the primary conclusions of the paper, which is the relation between signaling (RasGTP patches) and pseudopods and the implications for tip splitting.

The third term ensures that the cellular area *A* (which is the equivalent of the cellular volume in the 3D case) remains constant and can be viewed as an effective pressure. Finally, the last term represents an effective drag force, proportional to the local velocity *v*, and determines the time a pseudopod continues to move after the protrusion force has vanished. This term also yields a limit on the maximal speed, so that a constant force in one direction results in a constant speed rather than an unrealistic constant acceleration. A complete list of the parameter values can be found in the Supporting Text S1. The evolution of each node is found by solving
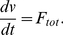
(5)


The entire simulation is performed in the following sequential steps: First, the reaction-diffusion equations (3) are solved on the entire membrane to find the value of the activator *a* at each point. Second, the force on each node is computed using Eq. (4). Finally, the nodes are advanced simultaneously according to Eq. (5). The time scale in the simulations can be converted to physical units by comparing the simulation cell speed to the cell speed obtained in the experiments and by taking a cell length that is comparable to the experimental dimensions of a cell. The internal compass in our simulations is updated every 2 minutes and additional computational details, including a description of adding and removing nodes, can be found in the methods section. A schematic diagram of the model cell as well as movies of several simulations can be found in the Supporting Information. The cell shape and motion both seem qualitatively realistic, and specifically, the formation, retraction and bifurcation of pseudopods resemble those seen in real cells.

### Simulation Results

**Figure 4:**
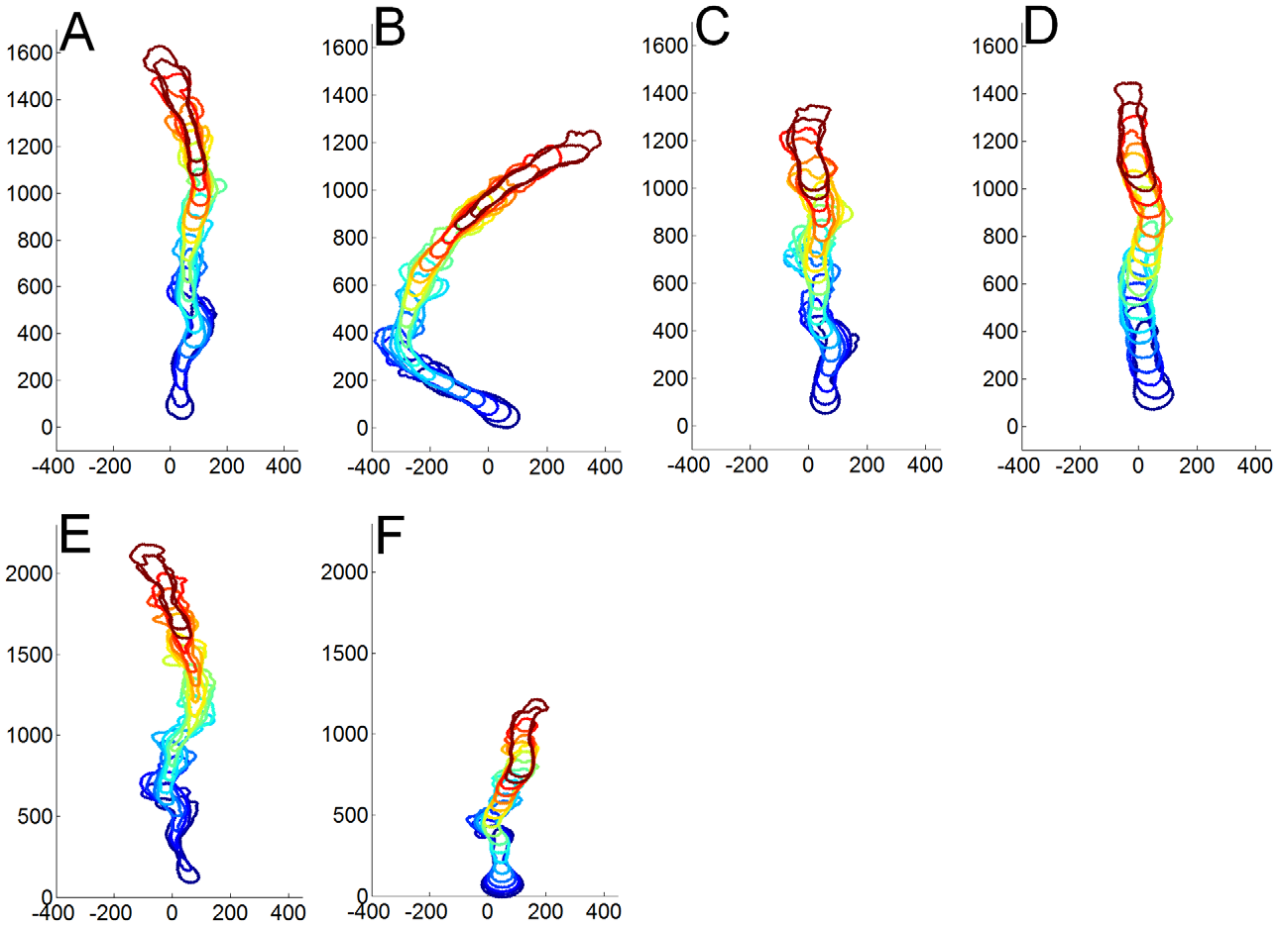
Simulation results. A time series of the motion of the model cell in an external upward gradient. Assuming a cell speed of 10 µm/min and a cell length of 20 µm, the time between successive frames is approximately 15 s. (a) Shallow gradient. Model parameters are: Spontaneous curvature *κ_0_* = 0.01, cortical tension *γ_1_* = 6.5 around the cell, *γ_2_* = 3.2 at the patch and *γ_3_* = 0.9 at areas of negative curvature (eq. (5)), polarization level *p* = 10 (eq. (S1)), friction *λ* = 0.1 (eq. (5)) and gradient width *σ* = 1 (eq. (2)). Other parameters are as given in the Table of Parameters in the Supporting Text S1. (b) Same as (a) with gradient width *σ* = 12. (c) Same as (a) with *p* = 4. (d) Same as (a) with *γ_1_* = 13 and *γ_2_* = 6.4. (e) Same as (a) with *λ* = 0.085. (f) Same as (a) with *λ* = 1.3.

Snapshots of typical simulation runs are presented in Figure 4 where the cell contour is tracked over time for various parameter sets. All the simulations presented in Figure 4a–f were run for the same time period but note the 25% difference in *y*-axes, the distance traveled by the cell, in Figure 4a–d versus Figure 4e–f. The computational cell in Figure 4a (also shown in video S3 in the Supporting Information) functions as a reference cell and has a CI of 0.966. Decreasing the gradient steepness, through a larger value of the parameter *σ* as in Figure 4b (and Video S4), leads to a smaller value of the CI (0.778), which is consistent with experimental results [44]. Figure 4c (and Video S5) shows a cell with a smaller value of the internal polarizability parameter *p* that determines the width of the excitable region along the membrane. This cell has a reduced speed, is less elongated than the reference cell of Figure 4a, but has only a slightly reduced CI (0.931), which is consistent with our observations for less developed cells as well other experimental data [7]. In Figure 4d, we show a trajectory of a cell with high cortical tension, parameterized by *γ_1_* and *γ_2_*. This cell exhibits fewer pseudopods but its speed is similar to that of Figure 4a and its CI is 0.954, which is very close to the CI of the reference cell. The model therefore predicts that the cortical tension does not strongly influence the CI of the cell, but does influence the frequency of pseudopod formation. In Figure 4e–f the magnitude of the friction parameter 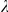 is varied, with low friction (Figure 4e) resulting in long pseudopods and increased cell speed and high friction (Figure 4f) resulting in shorter pseudopods and reduced cell speed.

**Figure 5:**
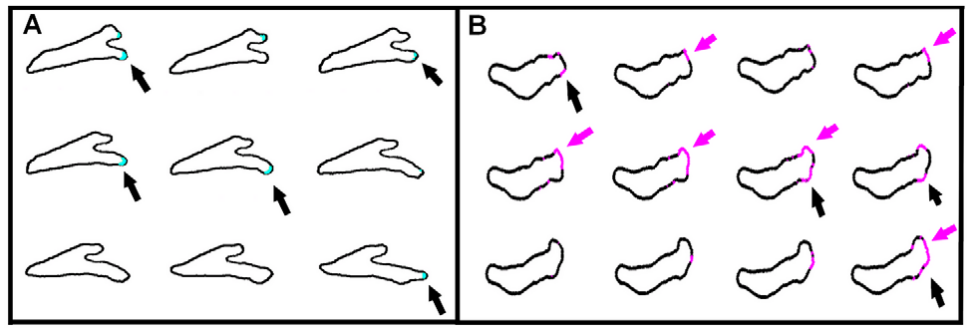
Patch dynamics. (a) Consecutive frames with a time interval of 6 seconds for a cell in the microfluidic device illustrating the come-and-go dynamics of the patches. The high-intensity patch appears (frame 1, top left), disappears (frames 2 and 3) and reappears (frame 4), as shown by the arrows. (b) The come-and-go dynamics of a patch in a simulated cell. The marked areas on the membrane (magenta) indicate a high *a*-field. The black and magenta arrows point on two patches, reappearing on different times.

**Figure 6:**
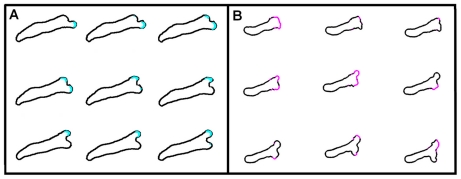
Apparent tip splitting (a) Experimental results of a pseudopod splitting in a cell in the microfluidic device. The new pseudopod is preceded by the appearance of a new high-intensity patch. Time gap between consecutive frames is 2 seconds. (b) Frames, separated by roughly 2.5 s, illustrating the apparent tip splitting in a simulation. The marked areas on the membrane (magenta) indicate a high *a*-field.

Our model is able to capture several qualitative features of *Dictyostelium* motility. For example, the experiments show that some pseudopods are maintained while others are retracted (Figure 5a). Pseudopods that are aligned with the gradient were found to have a higher probability of being maintained, and vice versa [30]. Interestingly, these maintained pseudopods exhibit “come-and-go” RasGTP patch dynamics in which a patch appears, disappears, and then re-appears, all at the same location, as can be seen in experiment and is shown in Figure 5a. This come-and-go patch dynamics is also observed in the results from our numerical simulations, as shown in Figure 5b. Furthermore, new pseudopods in our experiments are often created close to a previous one (Figure 6a), consistent with previous experimental studies where it was characterized as tip splitting [29], [30]. Importantly, our simulations also exhibit this tip splitting (Figure 6b), even though our model does not include any specific splitting mechanism.

**Figure 7:**
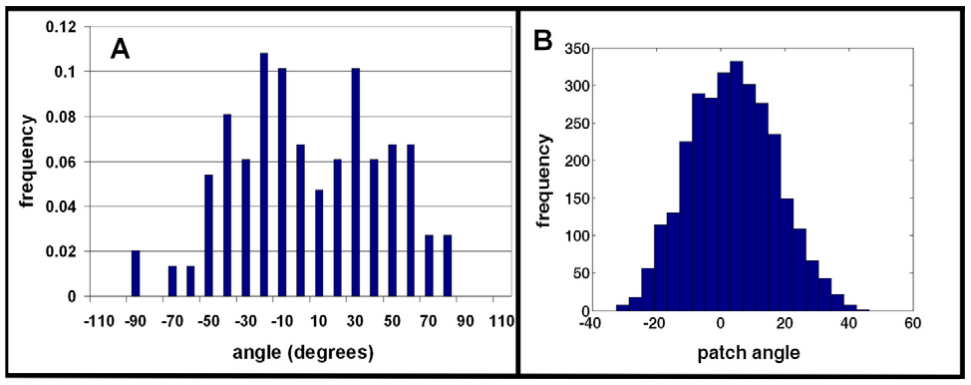
Simulation results. Pseudopod angle distribution (a) and patch angle distribution (b) for numerically generated cell data. The pseudopod angle distribution was analyzed using Quimp3.

To further investigate this apparent tip splitting behavior, we generated numerical cell data and analyzed these data using the same software as in previous experimental studies ([28], [29], [49] see Methods for more details). To this end, the locations of the membrane nodes were recorded and the contour of the simulated cells was computed using Matlab (Mathworks, Natick, MA). This cell contour was then used to create a full “cell body” by interpolating the discrete node locations and identifying the points that are inside the closed contour. The movement and shape of the simulated cell were analyzed using Quimp3 [49] to extract pseudopod statistics. The results are shown in Figure 7a. The angles of new pseudopods show a clearly bimodal distribution similar to that obtained by Bosgraaf et al. [29], implying the presence of a tip splitting mechanism. However, the distribution of patches that drive the membrane protrusions of the simulated cell, for the same numerical data set, is unimodal with a maximum at an angle that corresponds to the gradient direction (Figure 7b). The equations preclude a bimodal distribution of angles of new pseudopods resulting from this distribution of patches, since every pseudopod results from a patch. The apparent bimodal distribution must therefore be generated by the pseudopod detection algorithm.

**Figure 8:**
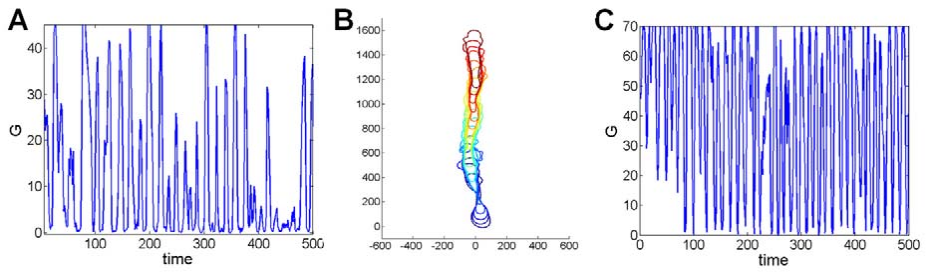
Global coupling effect. The global parameter *G* as a function of simulated time. (a) Simulation parameters as is Figure 4a. and *G(t = 0)* = 45. *G(t)* decreases as pseudopods grow and compete. (b) A cell track for a different set of parameters, in which the reaction-diffusion dynamics (eq. 2) is sped up and *G(0)* = 70. (c) *G(t)* for the same cell of (b). In this case the limiting role of *G* is less significant, yet the cell behavior and motion are virtually unchanged.

One important issue concerns the relative importance of the transient nature of the patch dynamics versus the global resource limitation in limiting the extensions of the pseudopods. Figure 8a shows *G(t)* for the simulation corresponding to the cell tracks shown in Fig. 4a. Clearly resource limitation is playing an important role and for this case the patch dynamics are mostly responsible for the *initiation* but not the cessation of protrusions. But, this is not necessary. In Fig. 8b we show a cell track example where we change the parameters of the chemical module to speed up patch dynamics (see details in the Supporting Text S1). As is seen in Figure 8c, *G(t)* oscillates but rarely dips down into the region where it limits protrusion; instead the patch dynamics is self-limiting. Altering the model in this manner does not change the aforementioned results regarding the response of the cell to varying the gradient strength and regarding the true source of apparent tip splitting seen in experimental studies.

## Discussion

Several recent studies have demonstrated that Ras activation is upstream of F-actin polymerization in a causal sequence leading to the formation of membrane protrusions and pseudopods [7], [22], [26], [27]. Our experimental assays on “2D” cells have been able to quantify the extent of spatial correlation between membrane areas of Ras activation (patches) and protrusions. We employed two different experimental assays to quantify this spatial correlation. In one set of experiments, we used the standard under-agar assay in which the vertical extent of cells was restricted by a thin layer of agar. We found a high spatial correlation between RasGTP patches and pseudopods in all analyzed cells, indicating that activated Ras and membrane protrusions occur at the same membrane location. However, drawbacks of this assay are that the vertical dimension is not known since cells are able to lift the agar to an unknown degree and that gradients are difficult to characterize. To overcome these shortcomings, we also performed experiments in microfluidic devices in which highly reproducible gradients with a well-defined direction and steepness are produced. Furthermore, the distance between the substratum and the roof of these devices is precisely specified (2 µm). We found that for cells in these devices the spatial correlation between patches and pseudopods was also large and comparable to the correlations found in the under-agar assay (Figure 2). This is also found to be the case for cells under uniform stimulation. Thus, the high spatial correlation between the patches and pseudopods appears to be insensitive to the details of the assay. We expect that this correlation is also large for cells that can extend freely in the vertical direction, however, this is difficult to determine since it requires a series of confocal scans in the vertical plane at each time point and significantly restricts the period of time a cell can be followed before suffering the effects of phototoxicity.

Establishing a high spatial correlation between the locations of active membrane regions and extending pseudopods is consistent with a causal relationship between the two and led us to create a model in which patches govern the location of membrane extensions. Our aim was to test how the dynamics of the signaling components influences the overall cell motility and shape dynamics. Our motility model addresses the two key ingredients, patch formation and pseudopod extensions, using two coupled modules that are responsible for obtaining realistic numerical cell shape and motion. First, the patch module is responsible for the creation of transient patches, as observed in the experiments. Second, the motility module incorporates a number of relevant forces acting on the cell membrane, including a term that couples the dynamic activator to the protrusive force. This modeling approach is distinct from previous attempts which mainly address specific stages of cell motility such as protrusion, adhesion or contraction [11], [33] or use a rule-based approach [12].

Our model, however, is still highly simplified. Since the biochemistry and specifically the exact reactions between the signaling components are still not fully known, the signaling module is mostly designed to replicate the experimental results so that the influence on the motility can be tested. We note that a recent paper by the Devreotes group introduces a very similar excitable medium approach [45]. Our motility module also simplifies a number of steps involved in generating membrane protrusions. For instance, the coupling between the patch module and the motility module, responsible for the protrusive force, is taken to be simply linear but may be more complex. Furthermore, the adhesion forces between the cell and the substratum are not explicitly modeled and are subsumed in the overall set of forces acting on the cell, as pushing forward is only possible in the presence of anchoring points. Also, a possible contribution from the bending energy is ignored. Despite these simplifications, our model is able to capture realistic cell behavior and shapes during chemotaxis (Figure 4), and provides insights into how dramatic changes in the cell shape can result from small changes in the signaling dynamics.

Our model contains a number of parameters that can be varied to mimic different experimental conditions. For example, the determination of the front of the cell in our model is a process that is subject to noise. This noise is taken from a distribution with width *σ* and the strength of the gradient can be adjusted by changing this width: a steep gradient corresponds to a narrow distribution (and small *σ*) while a shallow gradient corresponds to a wide distribution (and large *σ*). In agreement with experiments [44], [50], [51], we find that the CI is maximal for steep gradients and is reduced' for shallow gradients (Figure 4b).

Another parameter of the model, *p*, represents the cell's polarizability and controls the excitability change along the cell perimeter. As can be seen from Figure 4c, high values of this parameter lead to cells with a high CI, an elevated cell speed, and elongated cell shapes; this is the typical behavior of highly polarized *Dictyostelium* cells [7]. In contrast, low values of *p* result in rounder cells with a lower speed and lower CI, which is typical of cells in early developmental stages (see also Supporting Videos).

The cortical tension in our model is represented by the parameter γ, with high values of γ corresponding to a more rigid membrane. Not surprisingly, increasing the cortical tension leads to cell motion with fewer lateral pseudopods (Figure 4d). A direct comparison with experimental phenotypes is difficult since a quantification of the cortical tension in cells is problematic. However, it is commonly assumed that myosin is involved in establishing cortical rigor. Myosin mutants which have reduced cortical tension display more lateral pseudopods than wild-type cells and move more slowly [52], [53].

Recent studies of Andrew and Insall investigated chemotactic motion of *Dictyostelium* cells in the under-agar assay and presented evidence that new pseudopods were made in spatially restricted sites by splitting of the leading edge [30]. Furthermore, they found that pseudopods were generated at relatively constant intervals, independent of the orientation of the cell relative to the gradient, and that the survival and retraction of pseudopods were spatially controlled such that pseudopods aligned with the gradient were more likely to be maintained. They reasoned that their results contradict chemical compass models in which cells generate new pseudopods at the location of highest receptor occupancy (the needle of the compass) [31], [54]. They argued that cells guide their motion through a mechanism in which a new pseudopod splits off an existing one. A similar conclusion was reached by Bosgraaf and van Haastert, who analyzed a large number of pseudopodal extensions in chemotaxing *Dictyostelium* cells and found that the distributions of angles between the current and next pseudopod were bimodal with the peaks located at ±50° from the gradient direction [29].

Our model allows us to compare numerically obtained cell dynamics with these recent experimental observations. First of all, the reaction-diffusion model of the patch module is excitable and generates patches in a stochastic fashion. This guarantees that patches occur at rates that are set by the reaction-diffusion model and are independent of the cell's direction, consistent with experimental observations. Also, our model produces cell dynamics that resemble the tip splitting events observed in the experiments. In physical systems, tip splitting is a consequence of a spatial instability of the tip, resulting in the formation of multiple tips [36]. Such an instability, however, is not present in our model since an existing patch is stable, demonstrating that the observed events do not require an explicit tip splitting mechanism. The apparent tip splitting in our model is demonstrated in Figure 6b where a new patch appears close to the old one, leading to a new pseudopod that appears to split off from the old pseudopod.

The underlying patch dynamics can also explain the experimental observation that cells maintain pseudopods that are aligned with the direction of the gradient. As shown in Figure 5b, numerical patches can exhibit come-and-go dynamics characterized by the appearance of a patch, followed by its disappearance and re-appearance in roughly the same location. This repetitive patch formation at the same spatial location is more likely to occur in the direction of the gradient than away from the gradient. The accompanied membrane protrusion, however, does not necessarily exhibit this come-and-go dynamics, as protrusion initiation and cessation are smoothed and are not as abrupt as the upstream signaling. The time during which a pseudopod continues to move forward after the protrusive force has vanished is controlled in our model by the effective friction force parameter λ. This parameter represents the effective lag between the signal and its downstream response, for example due to the time needed for the process of actin polymerization. As a result, a series of consecutive but separate patches at the same location can lead to what looks like a “winning” single pseudopod.

It should be noted that in our model, the phenomenon of tip splitting results solely from the come-and-go dynamics of the patches, and is independent of other components of the model such as the global resource limitation, cortical tension and the specific form of the forces. All of these are needed for a realistic cell shape, but do not alter the main conclusions of our work, namely the effects of the signaling dynamics on the observed pseudopod behavior.

Pseudopods that are directed in the gradient direction and that have long apparent lifetimes can also underlie the experimentally observed bimodal distribution of pseudopod angles. Indeed, when we compared the pseudopodal angle distributions in numerical cell tracks using the automated software package Quimp3, we found a bimodal distribution even though the patch distribution exhibits a single peak in the direction of the gradient (Figure 7). Since our model does not contain an explicit tip splitting mechanism, and every pseudopod necessarily originates from a patch, this bimodality is purely an outcome of the algorithm, which detects a new pseudopod by identifying two spatially separated negative-curvature zones. The bimodal distribution produced by Quimp3 may result from undercounting pseudopods at zero angle and/or the elongated shape of cells.

In our model, the location of new pseudopods is determined by the location of patches, which are themselves controlled by the direction of the internal compass. The timescale for updating this internal compass is a parameter in our model and controls the persistence of the motion: a small timescale will lead to cells that change directions more often than cells with a larger timescale. Experimental values for this timescale, and how it depends on the external conditions, are presently unclear. The direction of the internal compass, and specifically its deviation from the external direction, is determined by the steepness of the gradient (Figure 3). For shallow gradients, the distribution of compass locations is wide while for steep gradients this distribution is narrow. As a direct consequence, we predict that the ratio between split and *de novo* pseudopods in shallow gradients is lower than in steep gradients. Experiments have only compared this ratio for cells in buffer and for cells in a gradient [29]. These experiments found, consistent with the above arguments, that the ratio is smaller for cells in buffer and extending this comparison for different gradient parameters would be interesting.

Our model contains a noisy internal compass with a direction that depends on the external gradient direction through our excitability parameter *β* and a noise level that is inversely proportional to the steepness of the gradient. In previous work, it was suggested that the generation of pseudopods at a constant rate and the generation of pseudopods in the “wrong” direction contradict the existence of such an internal compass [30]. However, our excitable reaction-diffusion system can produce patches at a constant rate. Furthermore, our results show that the internal compass model, even though it occasionally exhibits pseudopods directed in the wrong direction, is able to produce highly directed motion. Thus, our model is consistent with experimental results and indicates that cells might utilize an internal compass to direct their motion.

Since we want our cell simulator to behave in a robust manner even for more complex chemical driving fields, we have introduced several features of the motility module which do not appear to be essential for the case of primary interest here, namely motion in a stable, static gradient. Studies in which cells move in more complex environments, replete with obstacles and/or multiple sources, will be presented elsewhere; for those cases the global resource constraint is needed to ensure that eventually the cell moves in only *one* direction (see Supporting Figure S3) and the flexibility of negative curvature is needed (see Supporting Figure S4). We did not try to define a minimal model that would work only under more limited scenarios. Instead, our strategy was to embed patch dynamics in as realistic a motility module as we could infer from the data and then verify that our conclusions regarding cell shape, chemotactic index, and tip splitting were not affected by these more global considerations.

In conclusion, our model can capture several qualitative features of experimental cell motion. In particular, it is able to duplicate apparent tip splitting dynamics, apparent spatial control of pseudopod retraction, and the relatively constant rate of pseudopod formation. It is important to stress that these phenomena are produced without invoking a tip splitting mechanism suggesting that such a mechanism is not required in chemotaxing *Dictyostelium* cells. Furthermore, our model incorporates the notion of an internal compass which determines how the external gradient direction controls the locations of patches. Key in our model is the fact that our compass is subject to fluctuations. These fluctuations lead to a distribution of patches that is centered around the gradient direction but with a width that depends on the gradient strength. Thus, the needle of the compass is not necessarily pointing in the direction of the highest receptor occupancy at all times but fluctuates, leading to apparent tip splitting.

Based on the experimental results, our model connects the two major components in cellular chemotaxis, namely signaling and motility. We show that the dynamics of signaling molecules is related to the cell motility in a direct and localized manner, and this connection can explain a large amount of currently available data. This model was designed for the relatively simple system of *Dictyostelium* chemotaxis, but can also be extended to describe other types of cells such as immune cell migration, neuronal growth cone motility and cancer metastasis. We believe that highly interesting and valuable insights can be gained by focusing on the interplay between signaling and motility.

## Methods

### Experimental Assays

Microfluidic devices, originally designed and used to study gradient sensing in yeast [55], were modified to study cell migration in a “2D” environment by decreasing the height of the test chambers from 5 to 2 µm [35]. In brief, these devices consist of an array of parallel rectangular test chambers of various lengths between two flow channels that are 80 µm high. Continuous flow of buffer with zero or 100 nM cAMP in the flow channels creates stable linear gradients in the test chambers, with slopes determined by 100 nM/w, where w is the width of the chambers and varies from 100–650 µm.

Plasmid pDM115, a non-integrating vector containing the Ras binding domain of Raf1 tagged with GFP and driven by the actin15 promoter, was a gift from the van Haastert lab. Transformants of *D. discoideum* strain AX4 carrying this vector were selected for hygromycin or G418 resistance.

Exponentially growing cells were harvested from growth media by centrifugation, washed twice in KN2/Ca buffer (14.64 mM KH2PO4, 5.35 mM Na2HPO4, 100 µM CaCl2, pH 6.4), then resuspended at 5×10^6^ cells/ml and shaken for 5 hrs with 50 nM pulses of cAMP every 6 minutes to induce development Chemotaxis under agar was performed as previously described by Andrew and Insall [30]. Exponentially growing cells were also pulse-developed prior to loading into the microfluidic flow channel carrying buffer without cAMP. Cells were given 10 minutes to settle onto the coverslip prior to establishment of the gradient. Cells were imaged as they migrated across the test chambers. Fluorescent images (488 nm excitation) were captured every 2 seconds with a 63× oil objective on a spinning-disk confocal Zeiss Axiovert microscope equipped with a Roper Quantum 512SC camera. Images were collected using Slidebook 5 (Intelligent Imaging Innovations, Inc.).

### Experimental Analysis

For each frame, the contour of the cell was extracted and areas of cytosolic high intensity fluorescence were filtered out. Membrane areas of high intensity were detected using a threshold algorithm. The threshold value was adjusted to the movie characteristics, and usually taken to be within 10% difference from the maximal intensity. Protrusions were determined using the difference in membrane location between consecutive frames. Negative protrusions, i.e. inward motion of the membrane or retraction of a pseudopod, were filtered out in this analysis. In the case of several patches and several protrusions, the high-intensity points were clustered using the dendrogram algorithm [56] based on their Cartesian distances in space, so that well-distinguished, separate patches were obtained. The protrusion points were also clustered, and then each protrusion cluster was paired with the nearest patch (see Supporting Text S1).

### Computational Model

The cell membrane was parameterized by 100–200 nodes, conveniently stored as a double-linked list. The nodes represent the 1D membrane of the cell (see Figure S5 in the Supporting Information). To ensure sufficiently smooth variations of *a* along this membrane, we solved the reaction-diffusion equations (2) on a refined array of 5000 points. This was achieved by attaching a sub-array of points to each node in the membrane linked list.

The total number of membrane nodes is not constant and nodes are added and removed to keep the distance between them within a given range. When a pseudopod is extended, nodes are added at the tip where the membrane “stretches” and removed at the back of the cell. Care was taken such that the total amount of *a* and *b* remained constant during this reparametrization. Our default parameter set for the reaction-diffusion module and for the motility module is given in the Supporting Information.

The list of node locations was recorded every 500 iterations and used to construct the cell contour and the cell body. The cell was drawn using Matlab and the separate frames were constructed into a movie. This movie was later analyzed using Quimp3, an automated pseudopod-tracking algorithm [49].

## Supporting Information

Text S1 Additional information on correlation analysis, clustering, equation parameters, global coupling and model parameter values.
(DOC)

Figure S1 Angle difference analysis. (a) The analyzed cell with the identified RBD-GFP patch (cyan) and membrane protrusion (red), and their centers (marked in blue and yellow, respectively). (b) The angles *θ*_i_ and *ψ*_i_ of the patch and protrusion, respectively. The angles are measured with respect to the positive direction of the x-axis and the line connecting the center of the cell and the center of the patch or protrusion. (c) The cosine of the difference between the angles cos(*θ*_i_−*ψ*_i_) defines the spatial correlation between the patch and protrusion.
(TIF)

Figure S2 Excitability variance along the cell. The excitability parameter *β* as a function of the distance from the cell's front is shown for two values of the polarization parameter *p*. For *p* = 10 (red) the change is *β* is sharper than for *p* = 4 (blue), leading to a more polarized cell with higher chemotactic index and speed (see Figure 4 in the main text).
(TIF)

Figure S3 Global coupling effect. (a) Without global coupling – nonrealistic cell behavior. (b) With global coupling – realistic cell behavior that matches experimental evidence.
(TIF)

Figure S4The effect of low cortical tension at negative curvature. (a)–(b) A cell with two pre-defined patches, leading to two pseudopods. Cortical tension: 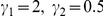 in both cases. (a) Cortical tension at negative curvature areas 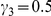, (b) With no curvature dependence of the cortical tension (i.e. tension is either 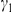 or 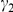, depending on the value of the activator *a* only). The cell in (a) exhibits a more biologically realistic shape compared to (b). (c)–(d) A cell with stochastically created patches, parameters as in Figure 4a in the main text. (c) with 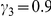, (d) with no negative-curvature dependence of the cortical tension. The cell in (d) is unable to produce significant pseudopods compared to the cell in (c).(TIF)

Figure S5 A schematic representation of the model cell. The membrane is represented by the nodes (large circles) while the reaction-diffusion equations are solved on the finer grid of points (smaller circles).
(TIF)

Video S1 A cell subjected to two chemoattractant sources, without global coupling.
(AVI)

Video S2 A cell subjected to two chemoattractant sources, with global coupling.
(AVI)

Video S3 Steep gradient (*σ* = 1 in eq. (3), polarization parameter *p* = 10 in eq. (S1)).
(AVI)

Video S4 Shallow gradient (*σ* = 12 in eq. (3), polarization parameter *p* = 10 in eq. (S1)).
(AVI)

Video S5 Less polarized cell: *p* = 4 (*σ* = 1).
(AVI)
