# Supplementary material for: Activated Membrane Patches Guide Chemotactic Cell Motility
Source: PLoS Comput Biol. 2011 Jun 30;7(6):e1002044. doi: 10.1371/journal.pcbi.1002044 (PMC3127810; doi:10.1371/journal.pcbi.1002044)
Supplement: Text S1 — Additional information on correlation analysis, clustering, equation parameters, global coupling and model parameter values. (DOC) [file pcbi.1002044.s001.doc]

# Activated Membrane Patches Guide Chemotactic Cell Motility

**Supporting Information – Supporting Text S1**

Inbal Hecht^1,2^, Monica L. Skoge^3^, Pascale G. Charest^3^, Eshel Ben-Jacob^2^, Richard A. Firtel^3^, William F. Loomis^3^, Herbert Levine^1,4^, Wouter-Jan Rappel^1,4^

1) Center for Theoretical Biological Physics, University of California San Diego, La Jolla, CA 92093, 2) School of Physics and Astronomy, Raymond and Beverly Sackler Faculty of Exact Sciences, Tel-Aviv University, 3) Cell and Developmental Biology, Division of Biological Sciences, University of California San Diego, La Jolla, CA 92093, 4) Department of Physics, University of California San Diego, La Jolla, CA 92093

**1. Correlation analysis demonstration**

We first identify areas of elevated RBD-GFP (patches) on the membrane by filtering out cytosolic fluorescence. Also, isolated points are filtered out by counting the number of neighbors with high intensity and removing all points with less than 4 neighbors. Membrane protrusions are identified by comparing two consecutive frames, as described in the main text and shown in Error: Reference source not founda. Next, the center of the patch and the center of the protrusion are identified, and the angles between the positive direction of the x-axis and the lines connecting the center of the cell and the centers of the patch and protrusion are measured (Error: Reference source not foundb). The cosine of the angle difference represents the spatial correlation (Error: Reference source not foundc and eq. (1) in the text).

(c)

**2. Clustering**

In many of the analyzed frames, there are several distinct areas of elevated intensity, as well as several protrusions in different directions. Therefore, we split the high intensity points between separate patches, using a clustering algorithm based on the Cartesian distances between the points. We first clusterize using a distance threshold of 25 pixels (about 5 μm) and then ensure that the number of clusters is within the pre-defined limit. Based on the experimental data, the number of different clusters (i.e. distinct patches) is between 1 and 3. Similarly, the membrane protrusions are clustered into 1 to 3 different pseudopods. The groups of patches and protrusions are matched by their proximity, so that several patches can be matched with the same protrusion and vice versa.

**3. Functional form of *β***

As described in the main text, the excitability of the reaction-diffusion system is controlled by the parameter *β*. This is used to distinguish between the front and the back of the cell, so that the system is only excitable at the front. This polarization arises from the directional sensing module, which is not explicitly modeled in this work. In the simulation, *β* varies with the distance from the cell’s front according to:

$\beta(x)=1.5-\sin^{p}\left(\frac{\pi}{2}\frac{L-d(x)}{L}\right)$ (S1)

where *L* is the front-back distance and *d(x)* is the distance of point x on the boundary from the front. The parameter *p* determines how abruptly $\beta$ changes with the distance from the cell front, as is demonstrated in Error: Reference source not found. Therefore, *p* has an impact on the width of the excitable zone on the membrane, and as we demonstrate in the main text, affects the polarization and shape of the cell as well as its locomotive properties.

**4. Protruding force: Local and global coupling**

The main novelty of this work is the coupling between the signaling process and the cellular motility. In our model, membrane signaling is represented by the patches of activation, which are areas of high *a* concentration. These patches govern the protruding force in the following form:

$f_{p}(a)=C(a-a_{0})\cdot\theta(G)$ (S2)

where *a_0_* is the activation threshold, *C* is a constant and $\theta(G)$ is a step function depending on a resource variable *G,* which is described below. While more complicated forms are possible, the complexity of the pathway from Ras activation to F-actin polymerization makes it impossible at this stage to exactly model the functional dependence between the two. Therefore, we decided to use the simplest possible relation.

The local dynamics of forces is coupled to the global dynamics of cellular biochemical components. Polymerization of F-actin is governed by the presence of various components, such as G-actin monomers and various enzymes, which diffuse in the cytosol. Different pseudopods are therefore coupled via these shared components, and a growing pseudopod in one area of the cell can effectively inhibit another pseudopod in a distant area by depleting the needed resources. We will describe our equation using the assumption that G-actin is a limiting resource for the F-actin needed for protrusion, but our model is general enough to encompass other logical possibilities as well. The dynamics of shared components is given by the following set of discrete equations, assuming rapid diffusion in the cytosol:

$\begin{matrix} \frac{\Delta F_{i}}{\Delta t}=\Gamma v_{i}\Delta l_{i}-\delta_{f}F_{i} \\ \frac{\Delta G}{\Delta t}=-\sum_{i} \Gamma v_{i}\Delta l_{i}+\sum_{i} \delta_{f}F_{i} \end{matrix}$ (S3)

where $F_{i}$ is the membrane bound F-actin at node *i*, *G* is the shared, freely diffusing component (e.g. G-actin), Г is a constant, $v_{i}$ is the membrane speed at node *i* and $\Delta l_{i}$ is the membrane length at node *i*, calculated by half the distance from node *i-1* to node *i+1*. The parameter values are given in the table below. The dynamics of *G* is coupled to the protruding force (eq. S2), which is non-zero only when *G*>0, namely when there are sufficient resources to allow further membrane protrusion.

The role and necessity of the global coupling are clear in the case of a cell exposed to two chemoattractant sources in different directions. In this case the cell will first produce two protrusions, but eventually will choose one of them and retract the other. This behavior can be seen, for example, in the work of Andrew and Insall [1]. To account for this mechanism in the model, we need to assume a global restricting mechanism, or an effective competition between different pseudopods in different areas of the cell, as can be seen in Error: Reference source not found and movies S1 and S2. While this is an extreme case and in most cases the global coupling is not as significant, we wanted the model to capture as wide range of the biology as possible and to be adequate for different cases and circumstances and therefore included this mechanism.

**5. Negative curvature**

As described in the main text, we found that in order to obtain realistic cells shapes, and specifically to get pseudopods with a large aspect ratio, we need the cortical tension $\gamma_{3}$of negative curvature areas to be smaller than the cortical tension at positive curvature areas. In the main text we also suggest a possible explanation for the origin of this effect. In Error: Reference source not found we show the two cases, with and without low $\gamma_{3}$, for two types of simulations: one with manually pre-assigned patches, and one with stochastic patches. In both types, the cell with low $\gamma_{3}$ has a shape that looks more realistic.

**6. Model cell**

The two modules of our model demand two different grids. While the motility module can be solved on a coarse grid, the reaction-diffusion system needs a much finer grid, due to the length and time scales determined by ε. To save computation time, we combined the two modules using the two types of grids. In the simulation, the membrane is constructed by a set of nodes, stored in a double-linked list. For each node, an array (of a variable length) includes the points of the finer grid, as shown in Error: Reference source not found . The reaction-diffusion equations are solved on the set of points, and then the values of *a* on the nodes are used for the motility calculation.

**7. Parameter values**

| ε  | 0.008 |
| --- | --- |
| μ | 0.5 |
| D_a_ | 0.05 |
| D_b_ | 0.25 |
| γ1 | 6.5 |
| γ2  | 3.2 |
| γ3 | 0.9 |
| λ | 0.1 |
| C | 2.5 |
| a_0_ | 0.7 |
| δ_f_ | 0.1 |
| G(t=0) (initial G-actin) | 45.0 |
| Г | 18 |
| Time steps ratio (reaction-diffusion dyanmics and motility) | 0.25 |

**8. Other parameters**

As shown in Fig. 8 in the main text, one can get qualitatively similar results with other sets of parameters. In Fig. 8b we used C=2.9 and the time step of the reaction-diffusion equations (2) was increased by a factor of two.
