# Supplementary figures and images for: Activated Membrane Patches Guide Chemotactic Cell Motility
Source: PLoS Comput Biol. 2011 Jun 30;7(6):e1002044. doi: 10.1371/journal.pcbi.1002044 (PMC3127810; doi:10.1371/journal.pcbi.1002044)

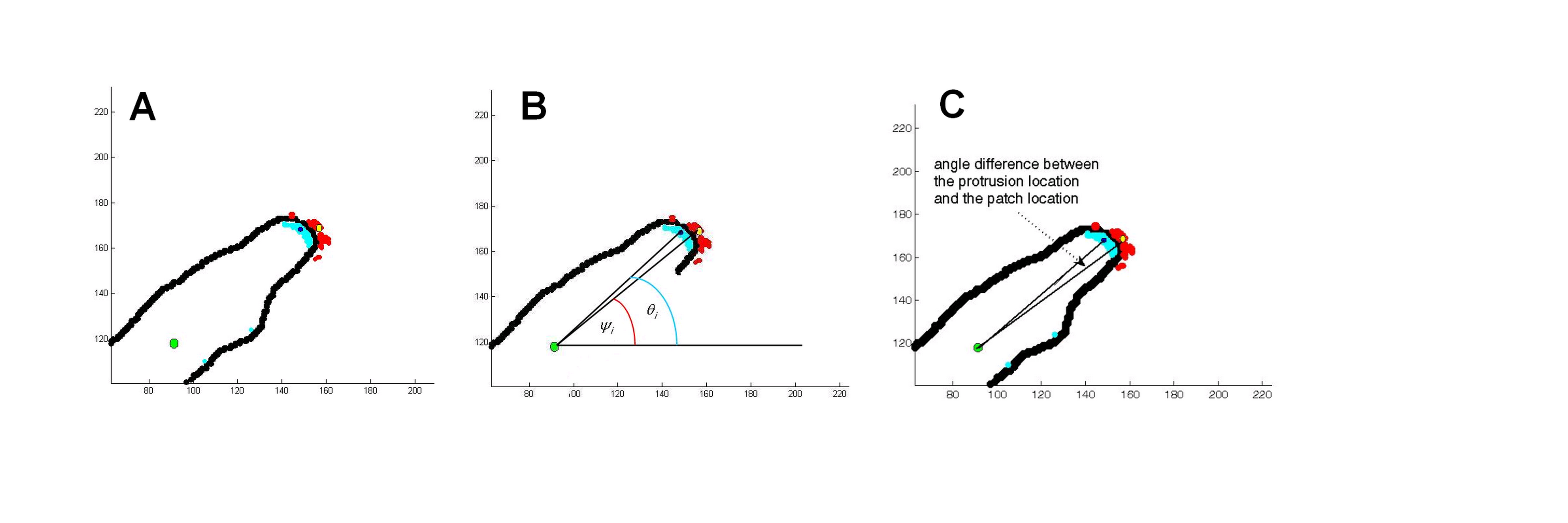

Supplement: Figure S1 — Angle difference analysis. (a) The analyzed cell with the identified RBD-GFP patch (cyan) and membrane protrusion (red), and their centers (marked in blue and yellow, respectively). (b) The angles θ i and ψ i of the patch and protrusion, respectively. The angles are measured with respect to the positive direction of the x-axis and the line connecting the center of the cell and the center of the patch or protrusion. (c) The cosine of the difference between the angles cos(θ i−ψ i) defines the spatial correlation between the patch and protrusion. (TIF) [file pcbi.1002044.s002.tif]

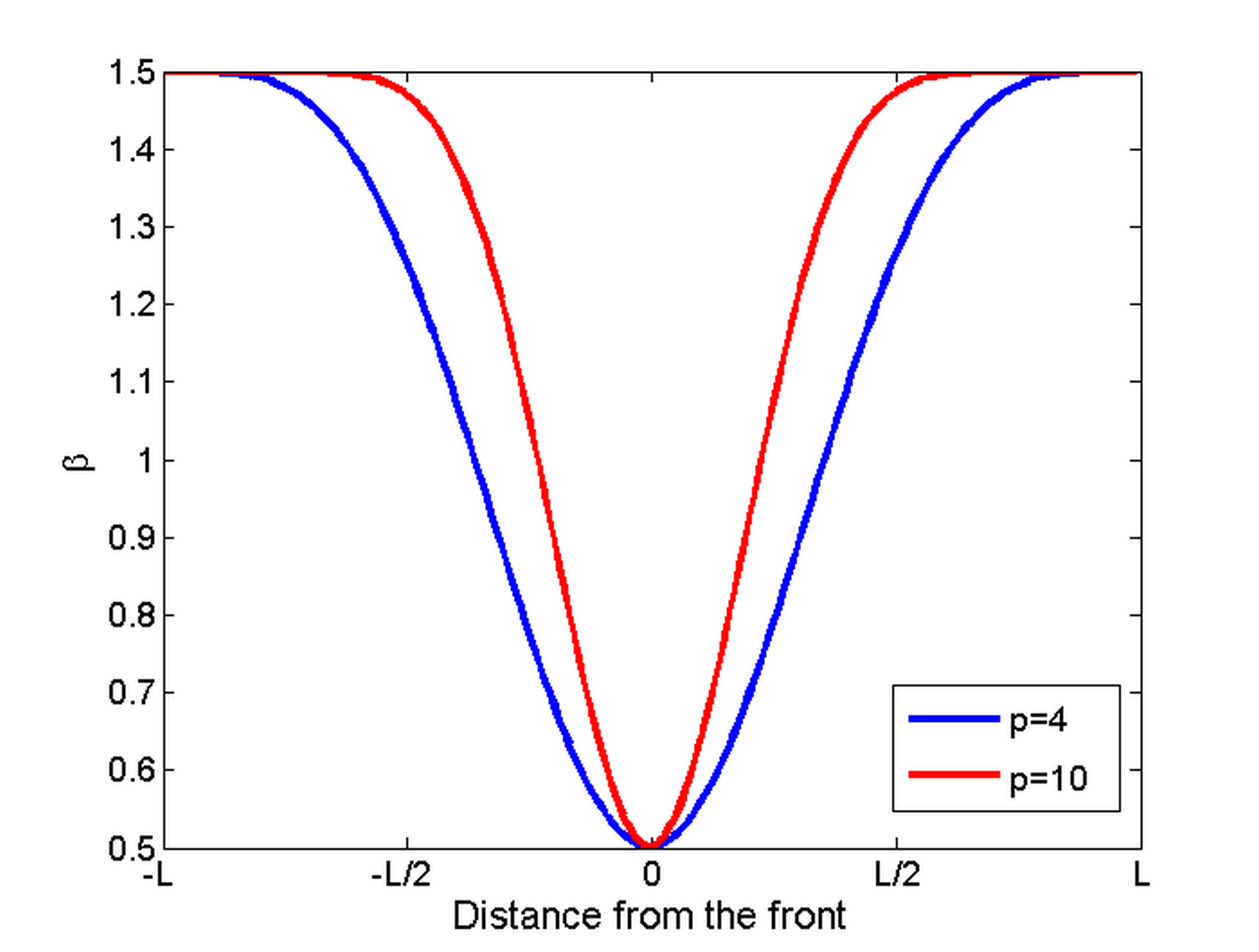

Supplement: Figure S2 — Excitability variance along the cell. The excitability parameter β as a function of the distance from the cell's front is shown for two values of the polarization parameter p. For p = 10 (red) the change is β is sharper than for p = 4 (blue), leading to a more polarized cell with higher chemotactic index and speed (see Figure 4 in the main text). (TIF) [file pcbi.1002044.s003.tif]

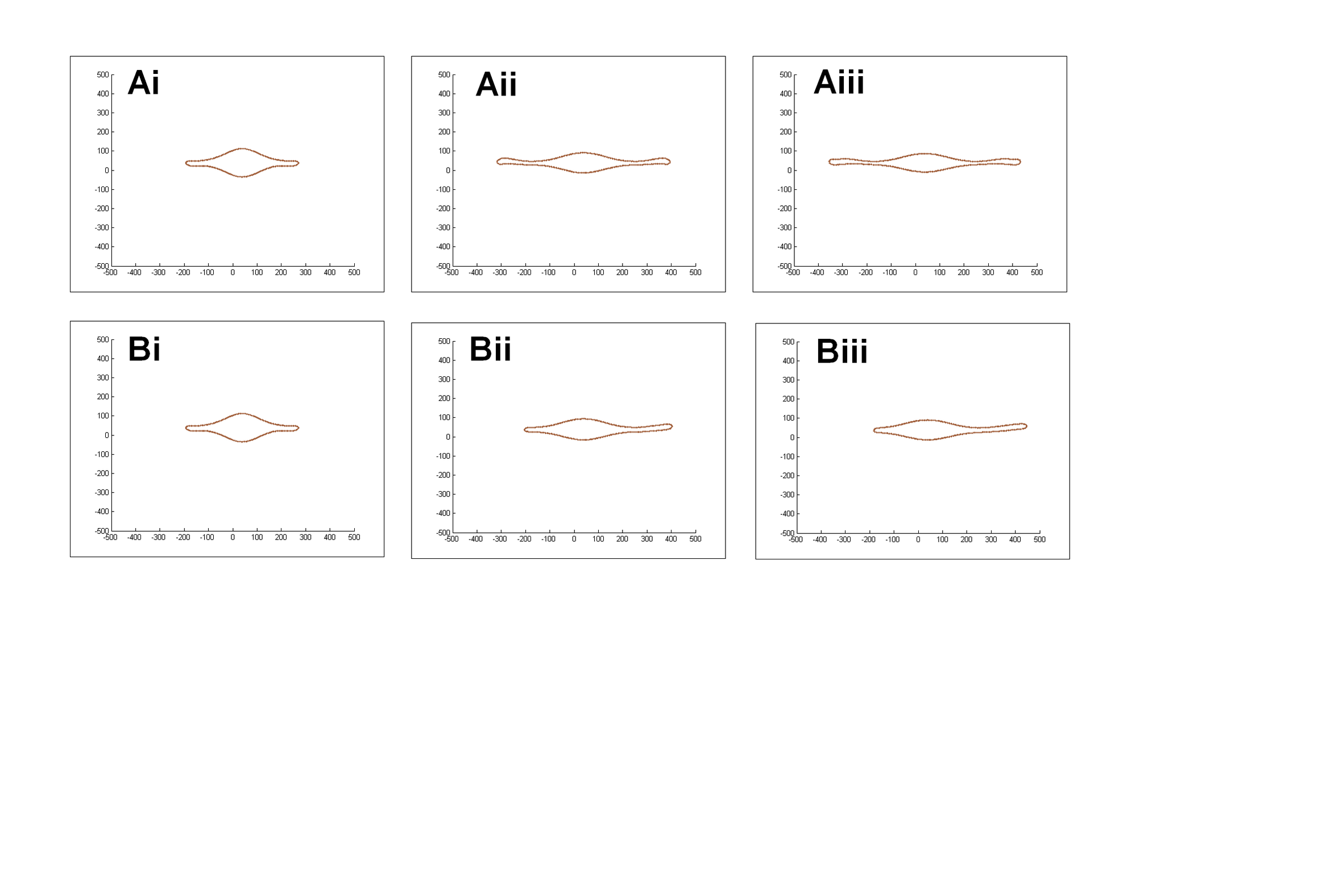

Supplement: Figure S3 — Global coupling effect. (a) Without global coupling – nonrealistic cell behavior. (b) With global coupling – realistic cell behavior that matches experimental evidence. (TIF) [file pcbi.1002044.s004.tif]

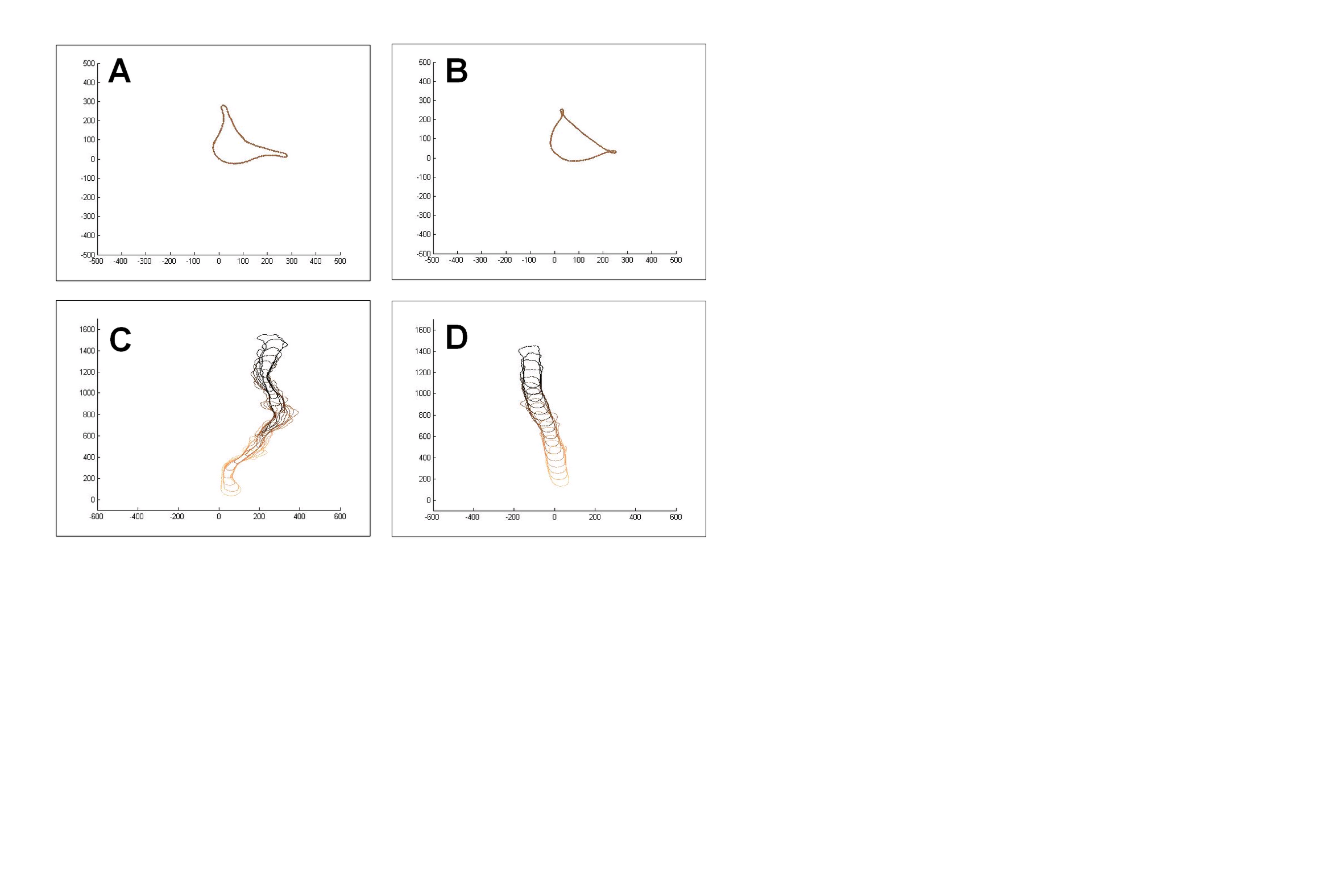

Supplement: Figure S4 — The effect of low cortical tension at negative curvature. (a)–(b) A cell with two pre-defined patches, leading to two pseudopods. Cortical tension: in both cases. (a) Cortical tension at negative curvature areas , (b) With no curvature dependence of the cortical tension (i.e. tension is either or , depending on the value of the activator a only). The cell in (a) exhibits a more biologically realistic shape compared to (b). (c)–(d) A cell with stochastically created patches, parameters as in Figure 4a in the main text. (c) with , (d) with no negative-curvature dependence of the cortical tension. The cell in (d) is unable to produce significant pseudopods compared to the cell in (c). (TIF) [file pcbi.1002044.s005.tif]

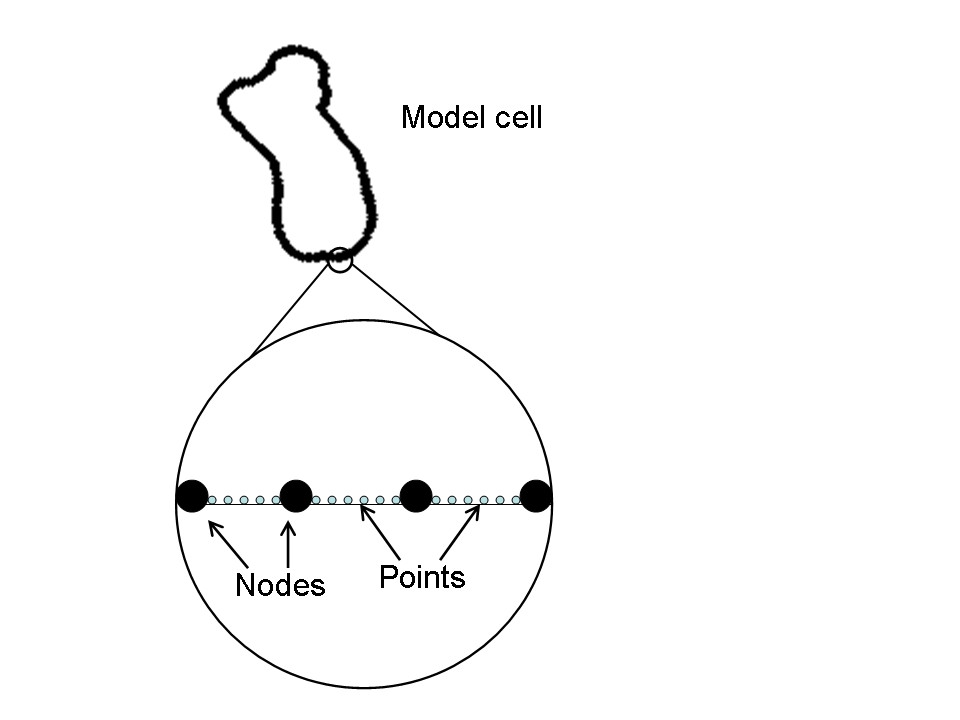

Supplement: Figure S5 — A schematic representation of the model cell. The membrane is represented by the nodes (large circles) while the reaction-diffusion equations are solved on the finer grid of points (smaller circles). (TIF) [file pcbi.1002044.s006.tif]
